# The Diversity of Seed-Borne Fungi Associated with Soybean Grown in Southern Poland

**DOI:** 10.3390/pathogens13090769

**Published:** 2024-09-06

**Authors:** Hanna Olszak-Przybyś, Grażyna Korbecka-Glinka

**Affiliations:** Department of Biotechnology and Plant Breeding, Institute of Soil Science and Plant Cultivation–State Research Institute, ul. Czartoryskich 8, 24-100 Puławy, Poland; gkorbecka@iung.pulawy.pl

**Keywords:** soybean, *Glycine max* (L.) Merrill, seed-borne fungi, storage fungi, fungal barcoding

## Abstract

Fungi have the potential to colonize soybean seeds in the field, during their maturation in the pods and after harvest, during storage. The aim of this study was to identify fungi inhabiting soybean seeds after storage with varying germination capacity and to evaluate their chemical composition. The research material consisted of twelve soybean seed lots collected from the fields in southern Poland and stored over winter. The germination percentage of these lots ranged between 20.67% and 81.33%. The seeds were subjected to analyses of the main chemical components and mycological analysis. Fungal isolates were subjected to taxonomic identification using microscopic methods and DNA sequencing (using internal transcribed spacer region and secondary barcoding regions). A total number of 355 fungal isolates from 16 genera were identified, with *Aspergillus*, *Alternaria*, and *Fusarium* being the most common. Species were successfully identified in 94% of isolates. Twelve examined seed lots varied significantly in the number of isolated fungal species (from 1 to 17). Moreover, they also differed in the isolated species composition. Highly significant positive correlation was found between the number of *Aspergillus psedudoglaucus* isolates and the content of free fatty acids. In turn, the number of *Fusarium* spp. isolates correlated negatively with protein and nitrogen content. Similarly, highly significant negative correlation was found between the number of all fungal isolates and the 1000-seed weight, indicating that smaller seeds are more vulnerable to fungal infection. The results obtained in this study identify species of fungi which may be responsible for lowering quality of the seeds obtained in southern Poland.

## 1. Introduction

Soybean [*Glycine max* (L.) Merrill.] is one of the most valuable oil crops. The global production of this crop in 2022 amounted to 348.8 million tons, which illustrates its enormous economic importance. The main soybean growers are Brazil (120.7 million tons), the United States (116.3 million tons), and Argentina (43.8 million tons). The production in Europe in 2022 amounted to 12.4 million tons, of which approx. 43.8 thousand tons were produced in Poland [1]. Soybean seeds are essential for producing oil or feed for livestock and are a valuable source of protein, oil, vitamins, and minerals for humans [2]. They contain 38% protein, 30% carbohydrates, 18% lipids, and 14% other substances [3]. They are used to produce soy milk, tofu, soy sauce, and paste, which are popular worldwide [4]. Soybean also plays a crucial role in various industries, as it is used in the production of biodiesel, pharmaceuticals, plastics, paints, and cosmetics. Due to its isoflavone content, soybean is believed to have anti-inflammatory, antioxidant, and antifungal properties. Furthermore, fermented soy products have been demonstrated to possess anti-cancer and anti-diabetic properties, and beneficial effects on the cardiovascular system [4,5].

Soybean is threatened by various abiotic and biotic stress factors, which decrease soybean seed quality and yield [6]. According to Roy [7], 80 genera belonging to economically important pathogen groups are associated with soybean diseases. Among them, fungi are one of the most significant biotic factors that adversely affect soybean growth and yield by attacking plants and seeds during their growth and after harvest [2]. Over 150 different species of fungi can inhabit soybean seeds, potentially reducing the nutritional value of seeds and leading to the production of mycotoxins [8]. Fungi that colonize and damage seeds are traditionally divided into field fungi and storage fungi, based on their ecological requirements [9]. Fungi belonging to genera such as *Alternaria*, *Cladosporium*, *Cercospora*, *Diaporthe*, and *Fusarium* can invade seeds while still in the field, causing damage before harvest. However, their incidence in the seeds may be significantly reduced during storage [9,10].

Nevertheless, if these field fungi survive in the stored seeds used later as sowing material, they may pose a significant threat to germinating soybean seeds, seedlings, and mature plants. They can reduce seedling emergence in the field and cause many diseases. For example, *Alternaria* spp. infection may result in leaf spot, pod necrosis, and seed decay, while *Diaporthe* spp. may cause Phomopsis seed decay, pod and stem blight, and stem canker [8]. In turn, *Fusarium* spp. may lead to Fusarium seed rot, and pod and stem blight [8].

As storage progresses, the occurrence of field fungi decreases in seeds, while storage fungi begin to colonize the seeds, reducing seed vigor and germination rates [8]. A decline in seed quality is evident through a reduction in the protein and oil content, as well as a rise in the free fatty acid content [8,11]. The predominant storage fungi belong to the genera *Aspergillus* and *Penicillium*. The proportion of *Aspergillus* increases by 1.3% after six months of storage, and by 7.5% after eight months [10]. The quantity of *Penicillium* in the stored seeds also increases over time, especially after six months of storage [10]. Storage fungi from both genera contribute to seed spoilage and damaged embryos [9]. They may also cause a reduction in the germination level and adverse changes in the shape, color, and biochemical composition of seeds. Seeds with reduced germination capacity are not suitable for sowing. Some of the storage fungi have the ability to produce toxins that are harmful to humans and livestock [2,12,13]. Mycotoxins produced by *Aspergillus flavus*, *Aspergillus parasiticus*, and *Penicillium* spp. can make seeds unsuitable for human and animal consumption. The estimated losses caused by storage fungi amount to more than $20 million a year in the USA [8]. 

Traditional methods of identifying fungi by recording colony appearance, growth rate, and morphological characteristics can often be misleading due to hybridization, cryptic speciation, and convergent evolution [14,15,16]. Identifying fungi based on morphology can also be challenging, especially when fungal cultures produce a limited number of morphological structures (e.g., conidia) that can be used for identification, or when these structures exhibit highly variable features [16,17]. As a consequence, DNA sequence-based methods have been developed for identifying species. The internal transcribed spacer (*ITS*) is the most commonly sequenced marker for accurately identifying a wide range of fungi [18,19,20]. Although the *ITS* region is generally considered a suitable barcode for fungi, it is insufficient for some seed-borne fungi such as *Aspergillus*, *Fusarium* and *Penicillium*. For these genera, it is recommended to use one or more single-copy protein-coding genes for species-level identification [17,21,22,23].

Dominant fungi associated with soybean seeds may differ between geographical regions. For example, in Brazil, *Colletotrichum truncatum* was identified as the predominant species responsible for anthracnose on soybean. Seeds infected with this pathogen were widely recognized as a major source of disease spread [24,25]. On the other hand, Cortina [26] found that *Fusarium* was the dominant genus on soybean seeds, accounting for 80–90% of all isolated fungi, while *Colletotrichum* represented only 5–10%. Research from China reveals that the main fungi isolated from soybean seeds in China belong to the *Fusarium* genus, with *Fusarium fujikuroi* being the most prevalent species [27]. In North America, *Fusarium* species were frequently reported in soybean seeds. The study found that 33% of soybean seed samples were infected with these fungi, and they were isolated from seeds collected from 80% of locations in Kansas [28]. A survey in Turkey revealed that 88% of soybean seeds were contaminated with fungi. The infection rates of *Cladosporium* spp., *Fusarium* spp., *Penicillium* spp., and *Aspergillus* spp. were 42%, 38% 10%, and 0.9% in soybean seeds, respectively [2].

It is well known that infected seeds can be a source of infection in the field, and can affect seed germination and the development of the disease at a younger stage [8]. Even certified seeds are not guaranteed to be free from seed-borne pathogens [9,10]. Furthermore, soybean sowing material typically comes from stored seeds because several months separate harvest and sowing in the subsequent season. Mycological analyses of the seeds after storage allow for the identification of culturable pathogens that were able to thrive in the stored seeds and could potentially cause plant disease in the field. They also detect storage fungi that potentially affect the seed quality of sowing material. Increased knowledge about seed-borne fungi may be used to develop effective and sustainable practices to reduce the damage they cause.

Many studies have been carried out examining the occurrence of fungi on and in soybean seeds [2,25,26,27], but so far, no such studies have been conducted in southern Poland. In our previous work [29], we characterized fungi isolated from soybean plants grown in one location in the southeast of the country. The aim of this study was to characterize the fungal community in stored soybean seeds originating from multiple fields in southern Poland. To accomplish this, identification of fungi was performed using mycological analyses supported by DNA sequencing of recommended barcoding regions. Germination tests and analyses of chemical composition of the seeds allowed for determining the impact field and storage fungi on soybean seed quality.

## 2. Materials and Methods

### 2.1. Seed Collection

Twelve samples of soybean seeds (1.5 kg each) were provided by seed producing company Agroyoumis (Table 1) in 2022, along with the data on the initial (pre-storage) germination percentage (ranging from 40% to 95%). 

**Figure 1:**
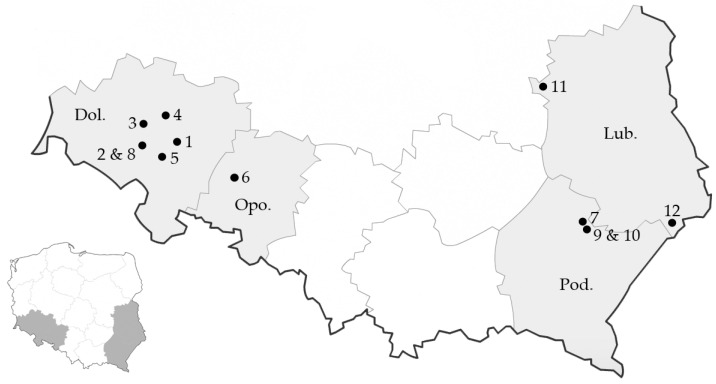
The map of four provinces of Poland with indicated locations of soybean fields where seeds analyzed in this study were collected in 2021 (locations numbered as in Table 1) The provinces are indicated by the following abbreviations: Dol—Dolnośląskie province, Opo—Opolskie province, Pod—Podkarpackie province, Lub—Lubelskie province.

These seed samples were collected in 2021 from 12 different soybean plantations designated for seed production. These were located in southern Poland in the following four provinces: Dolnośląskie, Lubelskie, Opolskie, and Podkarpackie (Figure 1). All samples were stored in the same warehouse (without temperature or humidity control) for approximately seven months, until April 2022, which is the typical time when soybean is planted in Poland. At that time, the collected seeds were included into our post-storage germination tests and other analyses described below.

### 2.2. Germination Test

The methodology used for seed germination assessment followed the guidelines of the International Seed Testing Association (ISTA) [30]. From every seed lot, three replicates of 100 seeds were tested. Seeds from each replicate were placed between moist filter paper rolls, covered with a plastic bag, and germinated in climatic chambers MC-1750 (Snijders Labs, Tilburg, The Netherlands) under the following conditions: 30 °C for 8 h in the light and 20 °C for 16 h in the dark, maintaining 90% humidity throughout. On the 8th day of the incubation period, the number of germinated seedlings was recorded, and the germination percentage was calculated for each seed lot.

### 2.3. Isolation and Morphological Identification of Fungal Species

One hundred soybean seeds (four replicates of 25 seeds) from each seed lot were used for mycological analysis. Soybean seeds were surface-disinfected with a 1.4% sodium hypochlorite solution for one minute, rinsed three times in sterile water, and dried on sterile tissue paper under airflow in Class II Microbiological Safety Cabinet (NordicSafe^®^, Esco, Singapore). Finally, the seeds were placed in Petri dishes containing Potato Dextrose Agar (PDA, Difco^TM^, Sparks, MD, USA) medium with tetracycline hydrochloride (2.5 mg L^−1^), in four replicates of 5 Petri dishes each. Five seeds were placed on each Petri dish and then incubated 20 °C for 14 days, with 8 h in the light and 16 h in the dark. The fungi that grew from seeds were isolated by cutting small pieces of the mycelium and transferring them to separate Petri dishes with PDA medium. Pure cultures of individual fungal isolates were used for taxonomic identification. The morphological characterization of the fungal isolates was carried out after incubation on PDA medium at 20 ± 2 °C. Isolates were first characterized by recording their morphological characteristics (color, size, shape; recorded for both sides of the culture) and microscopic features (shape and size of conidia and conidiophores, number of septa) using a NIKON Eclipse 80i microscope (Tokyo, Japan). Then, the recorded data were compared with species descriptions in taxonomic identification keys [31,32]. In this way, most isolates were identified at least to the genus level.

### 2.4. Molecular Species Identification of Fungal Isolates

DNA sequencing analysis was conducted to identify isolates that were indistinguishable at the genus level under the microscope, and to identify each isolate at the species level. For this purpose, genomic DNA was extracted using a modified CTAB method [33]. The extraction buffer contained the following: 3% *w*/*v* CTAB, 100 mM Tris-base, 20 mM EDTA, 1.4 M NaCl, pH = 8. The quality and quantity of the extracted DNA were assessed using a NanoDrop2000 (Thermo Scientific, Wilmington, DE, USA). All isolates were subjected to amplification and sequencing of the *ITS* region, a widely accepted fungal barcode. For this purpose, primers ITS1 and ITS4 were used [17,18,22]. However, the *ITS* region is often not variable enough to distinguish some closely related species in genera such as *Aspergillus*, *Fusarium*, and *Penicillium*. Therefore, the *Fusarium* isolates were identified at the species level by sequencing two highly informative genomic regions: the translation elongation factor (*TEF1*) and RNA polymerase second largest subunit (*RPB2*) [23]. For *Aspergillus*, calmodulin (*CaM*) was chosen as a recommended additional barcoding marker [17,34,35]. In the case of *Penicillium*, the beta-tubulin (*BenA*) gene region was used as a secondary marker [17,21,36]. 

The initial polymerase chain reaction (PCR) amplification for all studied regions (*ITS*, *TEF1*, *RPB2*, *CaM*, *BenA*) was performed in a volume of 25 µL containing 12.5 µL of Platinum Green Hot Start PCR 2× Master Mix (Invitrogen, Vilnus, Lithuania), 0.2 µM of each of the two primers, and 50 ng of DNA. The sequences of specific primers and thermal programs used for amplification of each of the five studied regions are provided in Table 2. PCRs were performed using a of C1000 thermal cycler (Bio-Rad, Singapore).

Subsequently, PCR products were treated with ExoSAP-IT reagent following the manufacturer’s protocol (Applied Biosystems, Vilnus, Lithuania) and then subjected to sequencing. The product of amplification of the *TEF1* region was sequenced using internal primers EF3 (5′-GTAAGGAGGASAAGACTCACC-3′) and EF22U (5′-AGGAACCCTTDCCGAGCTC-3′) [23]. The remaining three regions (*ITS*, *CaM*, *BenA*) were sequenced in both directions using the same primers as those used in the initial PCR. Cycle sequencing reactions were performed using Big Dye Terminator v3.1 chemistry (Applied Biosystems, Vilnus, Lithuania) following manufacturer’s recommendations and by means of a Veriti thermal cycler (Applied Biosystems, Singapore). The sequencing products were purified using ethanol/EDTA precipitation and separated on 3500 Genetic Analyzer (Applied Biosystems, Ibaraki, Japan). The sequences were reviewed and edited using Sequencing Analysis software v.6.0 (Applied Biosystems, Foster City, CA, USA). Then, forward and reverse sequences for each genomic region and each isolate were trimmed and assembled into continuous sequences using BioEdit 7.7.1. Representative sequences from each fungal species detected in each tested seed lot were deposited in GenBank. They were also subjected to a search of highly similar sequences in NCBI database (http://blast.ncbi.nlm.nih.gov accessed on 30 May 2024) by nucleotide BLAST. All *Fusarium* sequences were additionally compared to data in FUSARIUM ID v.3.0. database on the Galaxy platform (http://usegalaxy.eu/datasets/edit accessed on 31 May 2024) [37]. Species for each sequenced isolate was determined based on BLAST results with 99–100% identity.

### 2.5. Chemical Analysis of Soybean Seeds

The contents (%) of the following soybean seed components were measured: free fatty acids, total oil, protein, nitrogen, and moisture. In addition, the weight of 1000 seeds (1000-seed weight) was assessed for every seed lot. Free fatty acids and total oil content were analyzed by the accredited J.S. Hamilton analysis laboratory, following the established standards: PN-EN ISO 660:2010 (free fatty acids) and PN-EN ISO 659:2010 (total oil content). Meanwhile, protein, nitrogen, and moisture content were assessed by the Main Chemical Laboratory (GLACH, IUNG, Puławy, Poland), which is a research laboratory accredited by the Polish Accreditation Centre. The total nitrogen content in the seeds was determined by the Kjeldahl method, based on mineralization in sulfuric acid [38] and subsequent flow analysis with spectrophotometric detection. Then, protein content (CP) was calculated according to the following formula: CP = N × 6.25.

### 2.6. Statistical Analysis 

All data were processed using Excel (version 1808, Microsoft Office Standard 2019), whereas statistical analyses were carried out using software Statistica version 13.3 (Tibco Software, Palo Alto, CA, USA). From data on germination percentage and thousand-seed weight, averages and standard deviations were calculated for every seed lot. In order to compare germination percentage before and after storage, paired samples *t*-test was performed. Data averaged over three replicates were subjected to this test.

The non-parametric Spearman correlations were performed to assess the relationship between seed properties (thousand-seed weight, germination percentage, moisture content, nitrogen content, protein content, total oil content, and free fatty acid content) and the number of all fungal isolates obtained from 100 seeds. In addition, the same seed properties were correlated with the number of isolates belonging to genera and species accounting for at least 1% of all obtained isolates. Results of these analyses were reported in a correlation matrix for genera or species for which at least one correlation gave a significant result.

## 3. Results

**Figure 2:**
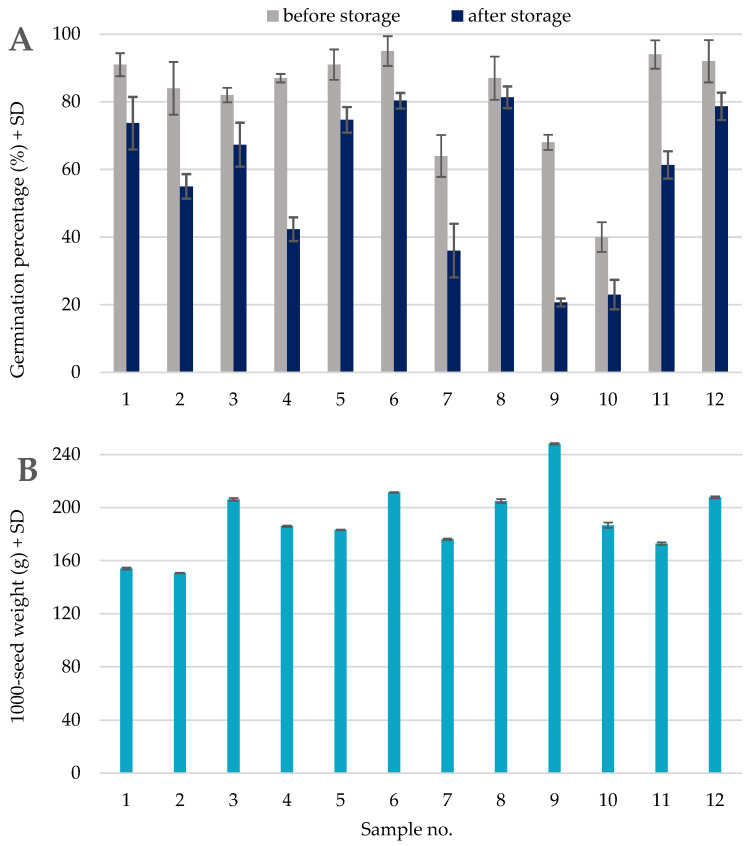
Germination percentage and 1000-seed weight of the twelve seed samples collected in four provinces in southern Poland: Dolnośląskie (sample nos. 1–5, 8); Opolskie (no. 6); Podkarpackie (nos. 7, 9–10); and Lubelskie (nos. 11–12). Values represent means of three replicates (±standard deviation). (**A**) Germination percentage of the seed samples determined before and after seven months of storage in the same warehouse. (**B**) Thousand-seed weight.

The germination percentage before storage ranged between 39.7 and 94.7% (average 81.1%), while after seven months of storage, it varied between 20.7 and 81.3% (average 57.9%; Figure 2). Paired samples *t*-test confirmed the negative impact of storage on seed germination. The difference in germination percentage assessed at the two time points was highly statistically significant (t = 6.07, df = 11, *p* < 0.001).

A total of 355 pure isolates were obtained from the twelve seed lots included in this study (Table 3). A combination of mycological analysis with sequencing of the recommended barcoding regions enabled the successful identification of 335 out of 355 isolates (94.4%) to the species level. The obtained DNA sequences were deposited in GenBank under the following accession numbers: PP873103–PP873174. The isolates were assigned to 48 species and 16 genera. The most frequently detected genera were *Aspergillus* (24.2% of all isolates), *Alternaria* (22.8%), *Fusarium* (16.3%), *Penicillium* (7.0%), *Cladosporium* (5.9%), *Botrytis* (4.8%), *Epicoccum* (3.9%), *Diaporthe* (3.4%), *Rhizopus* (2.8%), *Sarocladium* (2.8%), *Stemphylium* (2.0%), *Boeremia* (1.4%), and *Periconia* (1.4%). Each of the remaining genera, such as *Geomyces*, *Marquandomyces*, and *Phialophora*, accounted for less than one percent of all isolates obtained (Table 3). 

Most of the fungi identified in this study belonged to the *Aspergillus* (*As*) genus, as confirmed by micromorphology and DNA sequences of *ITS* and *CaM* regions. Seven *Aspergillus* species were successfully identified, with the most common being *As. pseudoglaucus*, representing 53 isolates, which constituted 14.9% of all isolates and 61.6% of *Aspergillus* isolates. The second most common species of *Aspergillus* was *As. flavus*, which constituted 7.3% of all isolates and 30.2% of *Aspergillus* isolates. Other *Aspergillus* species, including *As. montevidensis*, *As. niger*, *As*. *niveoglaucus*, *As. proliferans*, and *As. repens*, accounted for less than one percent of all isolates obtained in this study (Table 3).

The second largest genus identified was *Alternaria* (*Al*). It was mainly represented by *Al. alternata*, which constituted 71 isolates, comprising 20.0% of all isolates. *Al. infectoria* and *Al. tenuissima* were represented in less than one percent of all isolates, while other unidentified *Alternaria* species together accounted for 1.7% of all isolates (Table 3).

The next most common genus identified was *Fusarium*. A total of 58 isolates were found, accounting for 16.3% of all obtained isolates. Sequences of *TEF1* and *RBP2* regions enabled the identification of nine *Fusarium* species (Table 3). The most frequently isolated species was *F. graminearum* (32.8% of all *Fusarium* isolates), followed by *F. flagelliforme* (13.8% of all *Fusarium* isolates) and *F. avenaceum*, *F. equiseti*, and *F. tricinctum* (each representing 12.1% of all *Fusarium* isolates). Five or fewer isolates represented each of the remaining *Fusarium* spp.

The genus *Penicillium* was represented by nine species, distinguished based on *BenA* sequences. The most commonly detected *Penicillium* species was *P. brevicompactum* (27.0% of all *Penicillium* isolates). Within the *Cladosporium* genus, five different species were identified, of which *C. cladosporioides* was the most frequently detected. However, *ITS* sequences were insufficient to assign seven *Cladosporium* isolates to species.

**Figure 3:**
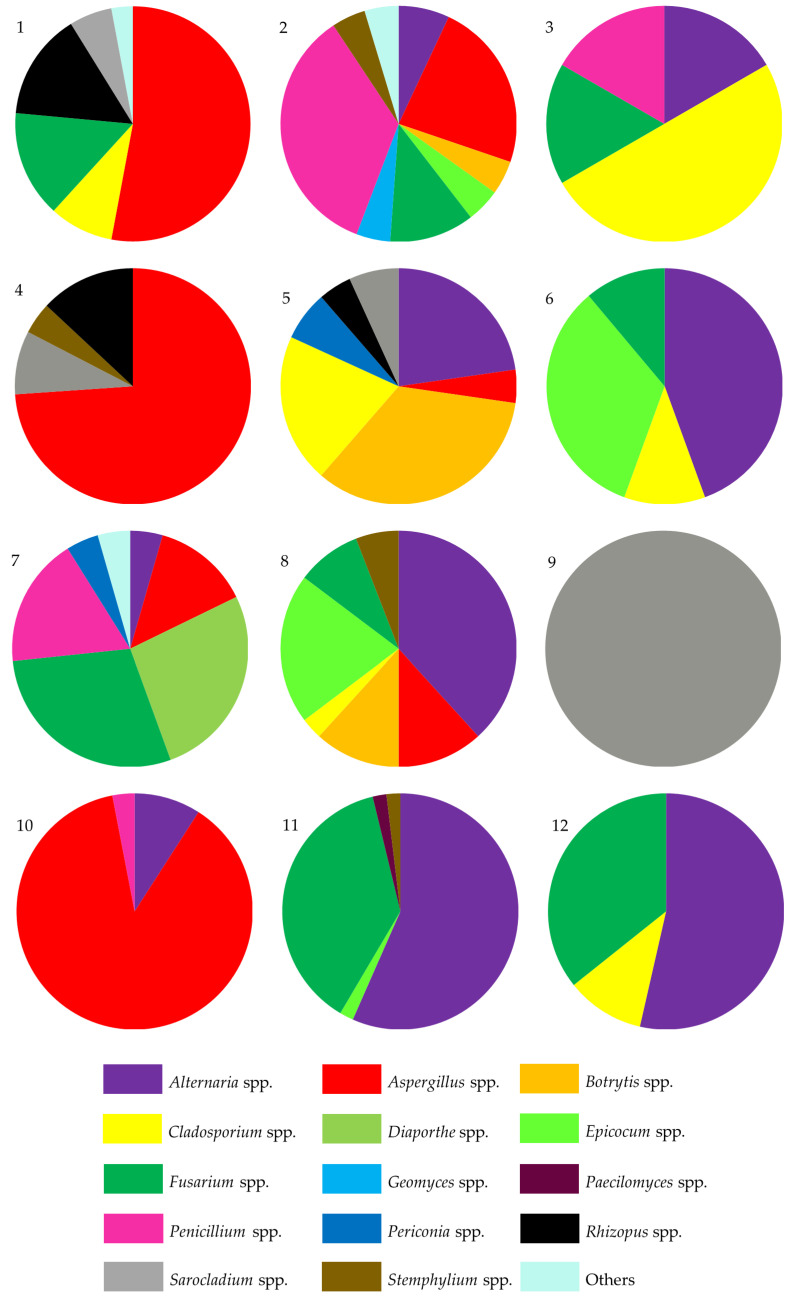
Pie charts showing the percentage of dominant fungal genera isolated from twelve soybean seed samples collected in the following four provinces in southern Poland: Dolnośląskie (nos. 1–5, 8); Opolskie (no. 6); Podkarpackie (nos. 7, 9–10); and Lubelskie (nos. 11–12).

Twelve seed lots included in this study differed with the frequency of isolated fungal species/genera. An overview at the genus level revealed differences in the predominant genera to which the fungal isolates belonged (Figure 3). Alternaria was the most commonly detected genus in seed lot nos. 6, 8, 11, and 12, representing 38.2% to 56.6% of all fungi detected in these samples. The *Aspergillus* genus was found most frequently in three seed lots, nos. 1, 4, 10, accounting for 52.9%, 73.9%, and 87.9% of the isolates, respectively. The predominant fungi isolated from the remaining seed lots, nos. 2, 3, 7, and 9, represented genera *Penicillium*, *Cladosporium*, *Fusarium*, and *Sarocladium*, respectively. 

The seed samples also differed in the number of isolated fungal species. From sample no. 9, only one species was isolated—*Sarocladium mali*—while the remaining eleven samples of soybean seeds were colonized by multiple species of fungi (Table S1). A low number of isolates obtained from sample no. 9 could have been caused by bacterial colonization of this seed sample (bacterial growth was observed around the seeds placed on the PDA medium despite the addition of antibiotics to the medium). The highest diversity was observed among fungi isolated from seed sample no. 2, as the isolates represented 17 species belonging to ten genera (Figure 3, Table S1).

Our study revealed statistically significant correlations between the number of *Aspergillus* or *Penicillium* isolates obtained and the percentage of free fatty acids in the seeds tested (Table 4). The content of free fatty acids in the seeds increases with the number of isolates of these storage fungi obtained from 100 seeds. 

Negative correlations were found between the protein and nitrogen content and the number of the obtained *Fusarium* isolates, in contrast to the number of *Sarocladium* isolates, which correlated positively with these seed properties. On the other hand, there was also a relationship between *Cladosporium* spp. and the higher percentage of germination (Table 4).

We also showed a statistically significant negative correlation between 1000-seed weight and the number of fungal isolates obtained. This result indicates that smaller seeds were more vulnerable to fungal colonization (Table 4).

## 4. Discussion

Numerous fungal pathogens that infect different parts of the soybean, such as stems, leaves, and roots, also can infect seeds. Not all of them are economically important, but most of them are widespread in the world. Many important soybean pathogens can be transmitted through seeds [8]. In this study, we isolated fungi from soybean seeds that belong to 16 different genera, including *Alternaria*, *Fusarium*, *Cladosporium*, *Botrytis*, *Diaporthe*, and *Boeremia*, which have been associated with soybean diseases such as Alternaria leaf spot, pod necrosis, seed decay, Fusarium seed rot, damping-off, and pod and steam blight [8,39]. These diseases contribute to reduced yield and economic losses. In addition, changes in the appearance of seeds caused by fungi such as *Alternaria*, *Colletotrichum*, and *Diaporthe* can reduce their market value [8].

More than one pathogen may infect the same seed and cause a mixture of symptoms [8]. The vast majority of seed samples analyzed in our study were infected with several genera of fungi. Our results are supported by numerous studies demonstrating the high diversity of fungi isolated from soybean seeds [7,8,39]. However, we also found significant differences in the composition and frequency of fungal species isolated from different seed lots.

In the temperate climate zone, soybean sowing material must be stored for several months between harvest and sowing in the next growing season. Soybean seed quality (germination capacity and chemical composition) may deteriorate during storage due to several biotic and abiotic factors [40]. The germination capacity of the samples analyzed in this study decreased by 6–48% during storage (Figure 2). Before storage, the germination percentage of nine samples exceeded 80%, the required threshold for the seeds used as sowing material. After storage, only two samples met this requirement. Therefore, our results are consistent with previous findings of decreased seed germination due to storage [41]. In the case of samples analyzed in this study, the loss of germination ability can be caused by seed-borne fungi and other microorganisms such as bacteria. The presence of bacteria in the seeds was the most evident in the case of sample no. 9, but we cannot exclude the possibility that other seed samples could also be infected. Moreover, cultivars may differ in their storability because of various genetically determined factors (such as chemical composition of the seeds and resistance to pathogens). 

Fungi affecting the quality of stored seeds belong to the genera *Aspergillus*, *Penicillium*, *Fusarium*, and *Alternaria*. *Aspergillus* species are dominant and play an important role in the seed biodeterioration [11]. Previous studies have shown that the genus *Aspergillus* is associated with reduced carbohydrate, protein, and oil content in stored seeds [42]. Furthermore, it causes an increase in free fatty acid content, which is considered a measure of seed deterioration [11]. *Aspergillus* species can produce lipases that hydrolyze triglycerides to free fatty acids and glycerol, which increases free fatty acid content [42]. Our study confirms the negative impact of the genus *Aspergillus* on soybean seed quality. We found a positive correlation between the number of *Aspergillus* isolates and the content of free fatty acids in the seed samples tested. Our results are in agreement with those reported by Dhingra [43]. Moreover, some *Aspergillus* species further reduce the quality of soybean seeds by producing mycotoxins, such as aflatoxins produced by *A. flavus* [44]. 

Next to *Aspergillus*, *Penicillium* is the most important genus of storage molds, and it is frequently isolated from soybean seeds. It causes discoloration, rotting, shrinking, seed necrosis, and toxification [7]. Some researchers have demonstrated that *Penicillium* species significantly reduced the fat content in soybean seeds [45]. Others point to a gradual increase in free fatty acid content in soybean seeds after *Penicillium* inoculation with an increasing storage period [42]. Our study also showed a positive correlation between the number of *Penicillium* isolates and free fatty acid content in the soybean seeds. These fungi may also negatively affect soybean seeds by producing mycotoxins [44]. 

In addition to typical storage fungi, other fungi such as *Fusarium* and *Alternaria* have been commonly isolated from soybean seeds [7,11,39]. In our study, *Alternaria* was the second and *Fusarium* was the third genus most frequently isolated from the soybean seeds tested, following the *Aspergillus* genus. *Alternaria* is generally considered to be a facultative parasite for soybean plants, and it can be detected in up to 95% of freshly harvested soybean seed samples [46]. During storage, *Alternaria* is gradually replaced by storage fungi, especially by different species of *Aspergillus* [47].

The cosmopolitan genus *Fusarium* is very diverse and includes many species. Some can attack soybean seedlings and mature plants as they grow in the field, causing sudden death syndrome, leaf necrosis, damping-off, and root rot [8,48]. Species such as *F. acuminatum*, *F. avenaceum*, *F. culmorum*, *F. equiseti*, and *F. oxysporum* are frequently isolated from soybean seeds and pods; they can reduce seed quality and vigor by reducing germination [7,27]. In general, fungi belonging to the *Fusarium* genus are recognized as field fungi that develop poorly on the seeds after long storage [49]. Carvalho et al. [10] reported a rapid drop in *Fusarium* incidence after the first two months of storage, after which the rate stabilized at a low level. We detected *Fusarium* as the third most frequent fungus on soybean seeds stored for eight months. This demonstrates the ability of these fungi to thrive in stored seeds. However, they not only thrive but also contribute to seed deterioration in storage. We found a negative correlation between the number of the obtained *Fusarium* isolates and the protein content (Table 3). Meriles et al. reported that *Fusarium* spp. infection of soybean seeds may lead to selective degradation of proteins [50]. These fungi can also reduce seed quality by production of mycotoxins, such as trichothecenes, which are cytotoxic to mammalian cells [44].

Soybean seeds may be colonized by fungi that benefit their quality. Our correlation analyses showed a potential positive effect of *Cladosporium* spp. on germination percentage. This could be explained by the fact that fungi belonging to this genus can produce gibberellins, hormones responsible for plant growth, especially those that stimulate seed germination [51]. In addition, *Cladosporium* spp. may act as antagonists, reducing mycelial growth of harmful fungi such as *Alternaria alternata*, *Botrytis cinerea*, *Rhizoctonia solani*, *Aspergillus*, *Fusarium*, *Penicillium*, and *Mucor* [51]. Interaction among different fungi coexisting in soybean seeds may be responsible for the positive effect of *Sarocladium* spp. on protein content in our samples of seeds. Only one of the four *Sarocladium*-containing samples also contained *Fusarium* (Table S1). Antagonistic interaction between *Sarocladium* and *Fusarium* fungi was demonstrated in other studies [52,53]. However, the effects of fungi on the seed properties suggested based on our correlation analyses and possible antagonistic interaction among the isolated fungi should be confirmed in additional experiments (e.g., seed inoculation and dual-culture plate assays). 

Our results show that seed-borne fungi can have a negative impact on the germination and quality of soybean seeds. In agricultural practice, these negative effects are mitigated by adequately drying the harvested seeds, selecting only well-germinating seeds for sowing, and using pre-sowing fungicide treatments. These procedures may not protect the sown seed fully [54]. Moreover, the rising prices of fungicides, their negative impact on the environment, and the development of pathogen resistance have stimulated searching for new methods of protecting soybean seeds. Thus, alternative, non-chemical methods have been developed. Physical treatments of the seeds using ultraviolet-C radiation, thermotherapy, or cold plasma have been shown to reduce the incidence of soybean seed-borne fungi [55,56,57]. Other studies demonstrated the antifungal effects of natural substances such as chitosan, plant extracts/powders, or essential oils [58,59,60,61]. Seed treatments with biocontrol agents such as *Trichoderma* spp. can also reduce the occurrence of fungal diseases in soybean seedlings [62,63]. The isolation of endophytes from soybean seeds and antifungal activity assays allow for the selection of microorganisms that can be used to reduce the impact of seed-borne pathogens [64]. Fungal cultures collected in this study can be used in future research.

## Figures and Tables

**Table 1:** List of soybean seed samples included in analyses in this study, cultivar, and geographic origin (province and field location).

No.	Cultivar	Province	Location ^#^
1	Lajma	Dolnośląskie	Jarząbkowice
2	Favorit	Dolnośląskie	Mierczyce
3	Kapral	Dolnośląskie	Lubin
4	Pompei	Dolnośląskie	Jemielno
5	Pompei	Dolnośląskie	Żarów
6	Orpheus	Opolskie	Buszyce
7	Madlen	Podkarpackie	Koziarnia
8	Kapral	Dolnośląskie	Mierczyce
9	Atlanta	Podkarpackie	Tarnogóra
10	Kapral	Podkarpackie	Tarnogóra
11	Bilyavka	Lubelskie	Stężyca
12	Mavka	Lubelskie	Lubycza Królewska

^#^ The location is shown on the map in Figure 1.

**Table 2:** Primers and PCR protocols used for initial amplification in the molecular identification of fungi isolated from soybean seeds.

Region	Genera of Fungi Amplified with This Region	Primer	Sequence (5′-3′)	Size (bp)	PCR Protocol Used in This Study	Ref.
Internal transcribed spacer (*ITS*)	All	ITS1 ITS4	TCCGTAGGTGAACCTGCGG TCCTCCGCTTATTGATATGC	~600	94 °C—5 min; 35 cycles (94 °C—1 min, 52 °C—1 min, 72 °C—1 min); 72 °C—10 min	[22]
Translation elongation factor 1-alpha (*TEF1*)	*Fusarium*	EF1 EF2	ATGGGTAAGGARGACAAGAC GGARGTACCAGTSATCATG	~700	94 °C—2 min; 35 cycles (94 °C—30 s, 56 °C—90 s, 68 °C—3 min); 68 °C—5 min	[23]
RNA polymerase second largest subunit (*RPB2*)	*Fusarium*	5f2 7cr	GGGGWGAYCAGAAGAAGGC CCCATRGCTTGYTTRCCCAT	~1700	94 °C—2 min; 35 cycles (94 °C—30 s, 56 °C—90 s, 68 °C—3 min); 68 °C—5 min	[23]
Calmodulin (*CaM*)	*Aspergillus*	CMD5 CMD6	CCGAGTACAAGGAGGCCTTC CCGATAGAGGTCATAACGTGG	~580	94 °C—1 min; 42 cycles (94 °C—1 min, 55 °C—30 s, 72 °C—90 s); 72 °C—10 min	[17]
Beta-tubulin (*BenA*)	*Penicillium*	Bt_2_a Bt_2_b	GGTAACCAAATCGGTGCTGCTTTC ACCCTCAGTGTAGTGACCCTTGGC	~500	94 °C—5 min; 35 cycles (94 °C—45 s, 55 °C—45 s, 72 °C—1 min); 72 °C—7 min	[21]

**Table 3:** Identity, BLAST result (GenBank ID and percent identity of the matching GenBank record), number, and percentage of fungi isolated from twelve samples of soybean seeds harvested in southern Poland.

Fungal Genus/Species ^#^	Top BLAST Result		Number (and %) of Isolates within Genus	Number (and %) of Isolates within Species
	GenBank ID *	Percent Identity		
***Aspergillus* spp.**			86 (24.2%)	
-*Aspergillus pseudoglaucus* Blochwitz	LT671276*^CaM^*	100%		53 (14.9%)
-*Aspergillus flavus* Link	OQ181323*^CaM^*	99.8%		26 (7.3%)
-*Aspergillus niger* Tiegh.	LC794815*^CaM^*	99.6%		2 (0.6%)
-*Aspergillus proliferans* G. Sm.	LT671146*^CaM^*	99.1%		2 (0.6%)
-*Aspergillus montevidensis* Talice & J.A. Mackinnon	OQ181343*^CaM^*	100%		1 (0.3%)
-*Aspergillus niveoglaucus* Thom & Raper	LT671255*^CaM^*	99.6%		1 (0.3%)
-*Aspergillus repens* (Corda) Sacc.	OR241663*^CaM^*	100%		1 (0.3%)
***Alternaria* spp.**			81 (22.8%)	
-*Alternaria alternata* (Fr.) Keissl.	MT373505*^ITS^*	100%		71 (20.0%)
-*Alternaria tenuissima* (Kunze) Wiltshire	MT212224*^ITS^*	99.8%		3 (0.9%)
-*Alternaria infectoria* E.G. Simmons	KR912323*^ITS^*	99.7%		1 (0.3%)
-*Alternaria* spp.				6 (1.7%)
***Fusarium* spp.**			58 (16.3%)	
-*Fusarium graminearum* Schwabe	OR689618*^TEF1^*	98.8%		19 (5.4%)
-*Fusarium flagelliforme* J.W. Xia, L. Lombard, Sand.-Den., X.G. Zhang & Crous	ON292364*^TEF1^*	99.8%		8 (2.3%)
-*Fusarium avenaceum* (Fr.) Sacc.	MK185024*^TEF1^*	100%		7 (2.0%)
-*Fusarium equiseti* (Corda) Sacc.	DQ842058*^TEF1^*	100%		7 (2.0%)
-*Fusarium tricinctum* (Corda) Sacc.	MG990939*^TEF1^*	100%		7 (2.0%)
-*Fusarium sambucinum* Fuckel (syn. *Fusarium cerealis*)	MH582259*^TEF1^*	100%		5 (1.4%)
-*Fusarium sporotrichioides* Sherb.	MZ078869*^TEF1^*	100%		3 (0.9%)
-*Fusarium fujikuroi* Nirenberg	OR933683^TEF1^	100%		1 (0.3%)
-*Fusarium redolens* Wollenw.	HQ731067*^TEF1^*	99.3%		1 (0.3%)
***Penicillium* spp.**			25 (7.0%)	
-*Penicillium brevicompactum* Dierckx	MK895703*^BenA^*	100%		7 (2.0%)
-*Penicillium aurantiogriseum* Dierckx	MN031407*^BenA^*	100%		4 (1.1%)
-*Penicillium polonicum* K.W. Zaleski	MK450898*^BenA^*	100%		4 (1.1%)
-*Penicillium griseofulvum* Dierckx	LC682861*^BenA^*	100%		3 (0.9%)
-*Penicillium melinii* Thom	KP016760*^BenA^*	99.8%		2 (0.6%)
-*Penicillium adametzii* K.W. Zaleski	JN625959*^BenA^*	100%		1 (0.3%)
-*Penicillium bialowiezense* K.W. Zaleski	MW980921*^BenA^*	100%		1 (0.3%)
-*Penicilium citrinum* Thom	OR241785*^BenA^*	100%		1 (0.3%)
-*Penicillium freii* Frisvad & Samson	OL631579*^BenA^*	100%		1 (0.3%)
-*Penicillium neoechinulatum* (Frisvad, Filt. & Wicklow) Frisvad & Samson	MN969388*^BenA^*	99.8%		1 (0.3%)
***Cladosporium* spp.**			21 (5.9%)	
-*Cladosporium cladosporioides* (Fresen.) G.A. de Vries	MN966603*^ITS^*	100%		9 (2.5%)
-*Cladosporium cucumerinum* Ellis & Arthur	OR008922*^ITS^*	99.7%		1 (0.3%)
-*Cladosporium pseudocladosporioides* Bensch, Crous & U. Braun	MF473221*^ITS^*	99.0%		1 (0.3%)
-*Cladosporium ramotenellum* K. Schub., Zalar, Crous & U. Braun	MT361323*^ITS^*	99.9%		1 (0.3%)
-*Cladosporium rectoides* Bensch, H.D. Shin, Crous & U. Braun	OQ165257*^ITS^*	99.8%		1 (0.3%)
-*Cladosporium uredinicola* Speg.	MW999339*^ITS^*	99.7%		1 (0.3%)
-*Cladosporium* spp.				7 (2.0%)
***Botrytis* spp.**			17 (4.8%)	
-*Botrytis cinerea* Pers.	MT250963*^ITS^*	100%		17 (4.8%)
***Epicoccum* spp.**			14 (3.9%)	
-*Epicoccum nigrum* Link	MN523199*^ITS^*	99.7%		14 (3.9%)
***Diaporthe* spp.**			12 (3.4%)	
-*Diaporthe novem* J.M. Santos, Vrandečič & A.J.L. Philips	MZ066809*^ITS^*	100%		6 (1.7%)
-*Diaporthe eres* Nitschke	MT573478*^ITS^*	100%		4 (1.1%)
-*Diaporthe phaseolorum* (Cooke & Ellis) Sacc. (syn. *Diaporthe caulivora*)	HM347679*^ITS^*	100%		1 (0.3%)
-*Diaporthe* sp.	HE774492*^ITS^*	99.5%		1 (0.3%)
***Rhizopus* spp.**			10 (2.8%)	
-*Rhizopus stolonifer* (Ehrenb.) Vuill.	OP437906*^ITS^*	100%		10 (2.8%)
***Sarocladium* spp.**			10 (2.8%)	
-*Sarocladium strictum* (W. Gams) Summerb.	OR346314*^ITS^*	99.8%		7 (2.0%)
-*Sarocladium mali* G.Y. Sun & Y.M. Hou	MF987662*^ITS^*	99.8%		3 (0.9%)
***Stemphylium* sp.**			7 (2.0%)	
-*Stemphylium vesicarium* (Wallr.) E.G. Simmons	MT629829*^ITS^*	100%		7 (2.0%)
***Boeremia* spp.**			5 (1.4%)	
*Boeremia exigua* (Desm.) Aveskamp, Gruyter & Verkley	MT397284*^ITS^*	99.5%		5 (1.4%)
***Periconia* spp.**			5 (1.4%)	
-*Periconia byssoides* Pers	MH859902*^ITS^*	99.6%		1 (0.3%)
-*Periconia pseudobyssoides* Markovsk. & A. Kačergius	LC014587*^ITS^*	99.8%		1 (0.3%)
-*Periconia* spp.				3 (0.9%)
***Geomyces* spp.**			2 (0.6%)	
-*Geomyces* sp.	JX270404*^ITS^*	99.5%		2 (0.6%)
***Marquandomyces* (syn. *Paecilomyces*) sp.**			1 (0.3%)	
*Marquandomyces marquandii* (Massee) Samson, Houbraken & Luangsa-ard (syn. *Paecilomyces marquandii*)	JN545822*^ITS^*	99.3%		1 (0.3%)
***Phialophora* sp.**			1 (0.3%)	
-*Phialophora* sp.	HQ713777*^ITS^*	99.6%		1 (0.3%)
**Total**			355	355

^#^ Species identity obtained from the top BLAST result and updated to the current name according to Index Fungorum, if necessary; * in the superscript, next to GenBank accession number, the abbreviated name of the sequenced region is indicated: *ITS*—internal transcribed spacer, *TEF1*—translation elongation factor 1-alpha, *Cam*—calmodulin, *BenA*—beta-tubulin.

**Table 4:** Correlation matrix between seed features and numbers of fungal isolates obtained from samples of 100 seeds. Values represent correlation coefficients, and they are marked with asterisks in case of statistical significance (* *p* < 0.05; ** *p* < 0.005).

Seed Features	Free Fatty Acid Content [%]	Total Oil Content [%]	Protein Content [%]	Nitrogen Content [%]	Moisture Content [%]	Germination [%]	1000-Seed Weight [g]
All fungi	0.25	0.33	−0.55	−0.55	−0.41	0.06	−0.80 **
*Aspergillus* spp.	0.59 *	0.33	−0.22	−0.22	0.11	−0.32	−0.57
*Aspergillus pseudoglaucus*	0.76 **	0.54	−0.56	−0.56	−0.21	−0.23	−0.40
*Fusarium* spp.	0.18	0.57	−0.81 **	−0.81 **	−0.51	0.23	−0.43
*Fusarium flagelliforme*	0.01	0.18	−0.59 *	−0.59 *	−0.24	−0.27	−0.38
*Penicillium* spp.	0.65 *	0.14	−0.41	−0.41	0.40	−0.45	−0.35
*Penicillium aurantiogriseum*	0.40	0.12	−0.65 *	−0.65 *	0.29	−0.33	−0.50
*Cladosporium* spp.	0.05	−0.03	0.26	0.26	−0.43	0.70 *	−0.07
*Sarocladium* spp.	−0.36	−0.48	0.59 *	0.59 *	0.08	−0.21	0.03

## Data Availability

Data are contained within the article and Supplementary Materials.

## Supplementary Materials

The following supporting information can be downloaded at https://www.mdpi.com/article/10.3390/pathogens13090769/s1, Table S1: Number and percentage of fungi isolated from individual seed lots.
