# Supplementary material for: The Diversity of Seed-Borne Fungi Associated with Soybean Grown in Southern Poland
Source: Pathogens. 2024 Sep 6;13(9):769. doi: 10.3390/pathogens13090769 (PMC11434997; doi:10.3390/pathogens13090769)
Supplement: Supplementary file 1 [file pathogens-13-00769-s001.zip › pathogens-3082513-supplementary.pdf]

Supplementary table

The Diversity of Seed-born Fungi Associated with Soybean Grown in Southern Poland

Hanna Olszak-Przybyś*, Grażyna Korbecka-Glinka

Department of Biotechnology and Plant Breeding, Institute of Soil Science and Plant Cultivation-State Research Institute, ul. Czartoryskich 8, 24-100 Puławy, Poland; gkorbecka@iung.pulawy.pl (G.K.-G.)

* Correspondence: holszak@iung.pulawy.pl

Table S1. Number and percentage of fungi isolated from individual seed lots.

Fungal species	Number (and percentage) of isolates obtained from 12 different samples of soybean seeds												
	**1**	**2**	**3**	**4**	**5**	**6**	**7**	**8**	**9**	**10**	**11**	**12**	**Total**
Alternaria alternata		3 (6.98%)	1 (16.67%)		10 (22.73%)	3 (33.33%)	1 (2.22%)	12 (35.30%)		3 (9.09%)	30 (56.60%)	8 (28.57%)	71 (20%)
Alternaria infectoria						1 (11.11%)							1 (0.28%)
Alternata unknown species							1 (2.22%)					5 (17.86%)	6 (1.69%)
Alternata tenuissima								1 (2.94%)				2 (7.14%)	3 (0.85%)
Aspergillus flavus		4 (9.30%)		16 (69.56%)			2(4.45%)	4 (11.76%)					26 (7.32%)
Aspergillus montevidensis				1 (4.35%)									1 (0.28%)
Aspergillus niger					2 (4.55%)								2 (0.56%)
Asergillus niveoglaucus										1 (3.03%)			1 (0.28%)
Aspergillus proliferans										2 (6.06%)			2 (0.56%)
Aspergillus pseudoglaucus	18 (52.94%)	6 (13.95%)					4 (8.89%)			25 (75.76%)			53 (14.93%)
Aspergillus repens										1 (3.03%)			1 (0.28%)
Boeremia exigua		1 (2.33%)						4 (11.76%)					5 (1.4%)
Botrytis cinerea		2 (4.65%)			15 (34.10%)								17 (4.79%)
Cladosporium cladosporioides	1 (2.94%)		1 (16.67%)		7 (15.91%)								9 (2.54%)
Cladosporium cucumerinum								1 (2.94%)					1 (0.28%)
Cladosporium pseudocladosporioides						1 (11.11%)							1 (0.28%)
Cladosporium ramotenellum			1 (16.67%)										1 (0.28%)
Cladosporium rectoides		1 (2.33%)											1 (0.28%)
Cladosporium unknown species	2 (5.88%)				2 (4.55%)							3 (10.71%)	7 (1.97%)
Cladosporium uredinicola			1 (16.67%)										1 (0.28%)
Diaporthe eres							4 (8.89%)						4 (1.13%)
Diaporthe novem							6 (13.33%)						6 (1.69%)
Diaporthe phaseolorum							1 (2.22%)						1 (0.28%)
Diaporthe sp.							1 (2.22%)						1 (0.28%)
Epicocum nigrum		2 (4.65%)				3 (33.33%)	1 (2.22%)	7 (20.59%)			1 (1.89%)		14 (3.94%)
Fusarium avenaceum							7 (15.56%)						7 (1.97%)
Fusarium sambucinum		5 (11.62%)											5 (1.4%)
Fusarium equiseti	5 (14.71%)		1 (16.67%)			1 (11.11%)							7 (1.97%)

Fungal species	Number (and percentage) of isolates obtained from 12 different samples of soybean seeds												
	**1**	**2**	**3**	**4**	**5**	**6**	**7**	**8**	**9**	**10**	**11**	**12**	**Total**
Fusarium flagellioforme							6 (13.33%)				2 (3.78%)		8 (2.25%)
Fusarium fujikuroi											1 (1.89%)		1 (0.28%)
Fusarium graminearum											15 (28.30%)	4 (14.29%)	19 (5.35%)
Fusarium redolens												1 (3.57%)	1 (0.28%)
Fusarium sporotrichioides												3 (10.71%)	3 (0.85%)
Fusarium tricinctum								3 (8.82%)			2 (3.78%)	2 (7.14%)	7 (1.97%)
Geomyces unknown species		2 (4.65%)											2 (0.56%)
Marquandomyces marquandii											1 (1.89%)		1 (0.28%)
Penicillium adametzii										1 (3.03%)			1 (0.28%)
Penicillium aurantiogreseum		3 (6.98%)					1 (2.22%)						4 (1.13%)
Penicillium bialowiezense		1 (2.33%)											1 (0.28%)
Penicillium brevicompactum							7 (15.56%)						7 (1.97%)
Penicillium citrinum			1 (16.67%)										1 (0.28%)
Penicillium freii		1 (2.33%)											1 (0.28%)
Penicillium griseofulvum		3 (6.98%)											3 (0.85%)
Penicillium melinii		2 (4.65%)											2 (0.56%)
Penicillium neoechinulatum		1 (2.33%)											1 (0.28%)
Penicillium polonorum		4 (9.30%)											4 (1.13%)
Periconia byssoides					1 (2.27%)								1 (0.28%)
Periconia pseudobyssoides					1 (2.27%)								1 (0.28%)
Periconia unknown species					1 (2.27%)		2 (4.44%)						3 (0.85%)
Phialophora unknown species	1 (2.94%)												1 (0.28%)
Rhizopus stolonifer	5 (14.71%)			3 (13.04%)	2 (4.55%)								10 (2.82%)
Sarocladium mali									3 (100%)				3 (0.85%)
Sarocladium strictum	2 (5.88%)			2 (8.70)	3 (6.82%)								7 (1.97%)
Stemphylium vesicarium		2 (4.65%)		1 (4.35%)			1 (2.22%)	2 (5.88%)			1 (1.89%)		7 (1.97%)
**Total no. of the obtained isolates**	**34**	**43**	**6**	**23**	**44**	**9**	**45**	**34**	**3**	**33**	**53**	**28**	**355**
**Number of the detected species**	**7***	**17**	**6**	**5**	**10***	**5**	**15***	**8**	**1**	**6**	**8**	**8***	

* Number of detected species can be underestimated because not all species were successfully identified.
